# Reorganization of Thalamic Inputs to Lesioned Cortex Following Experimental Traumatic Brain Injury

**DOI:** 10.3390/ijms22126329

**Published:** 2021-06-13

**Authors:** Xavier Ekolle Ndode-Ekane, Maria del Mar Puigferrat Pérez, Rossella Di Sapia, Niina Lapinlampi, Asla Pitkänen

**Affiliations:** A. I. Virtanen Institute for Molecular Sciences, University of Eastern Finland, FI-70211 Kuopio, Finland; mariadelmar.pupe@gmail.com (M.d.M.P.P.); rossella.disapia@marionegri.it (R.D.S.); niina.lapinlampi@uef.fi (N.L.); asla.pitkanen@uef.fi (A.P.)

**Keywords:** axonal injury, hyperexcitation, lateral fluid-percussion injury, perilesional cortex, thalamo-cortical projection, traumatic brain injury

## Abstract

Traumatic brain injury (TBI) disrupts thalamic and cortical integrity. The effect of post-injury reorganization and plasticity in thalamocortical pathways on the functional outcome remains unclear. We evaluated whether TBI causes structural changes in the thalamocortical axonal projection terminals in the primary somatosensory cortex (S1) that lead to hyperexcitability. TBI was induced in adult male Sprague Dawley rats with lateral fluid-percussion injury. A virus carrying the fluorescent-tagged opsin channel rhodopsin 2 transgene was injected into the ventroposterior thalamus. We then traced the thalamocortical pathways and analyzed the reorganization of their axonal terminals in S1. Next, we optogenetically stimulated the thalamocortical relays from the ventral posterior lateral and medial nuclei to assess the post-TBI functionality of the pathway. Immunohistochemical analysis revealed that TBI did not alter the spatial distribution or lamina-specific targeting of projection terminals in S1. TBI reduced the axon terminal density in the motor cortex by 44% and in S1 by 30%. A nematic tensor-based analysis revealed that in control rats, the axon terminals in layer V were orientated perpendicular to the pial surface (60.3°). In TBI rats their orientation was more parallel to the pial surface (5.43°, difference between the groups *p <* 0.05). Moreover, the level of anisotropy of the axon terminals was high in controls (0.063) compared with TBI rats (0.045, *p <* 0.05). Optical stimulation of the sensory thalamus increased alpha activity in electroencephalography by 312% in controls (*p >* 0.05) and 237% (*p >* 0.05) in TBI rats compared with the baseline. However, only TBI rats showed increased beta activity (33%) with harmonics at 5 Hz. Our findings indicate that TBI induces reorganization of thalamocortical axonal terminals in the perilesional cortex, which alters responses to thalamic stimulation.

## 1. Introduction

The cerebral cortex is the brain area most affected by traumatic brain injury (TBI) [1]. The ensuing cortical lesion ultimately leads to alterations in brain networks. These network alterations, mostly attributed to diffuse axonal injury and the less known axonal plasticity, underlie many of the post-TBI clinical outcomes [2]. Several clinical TBI studies have demonstrated the loss of axonal integrity in numerous white matter structures with direct consequences on the functional outcome [3,4,5]. Our understanding of the post-TBI network reorganization and the consequent effects on the clinical outcome remains limited.

Preclinical models of TBI are extensively used for elucidating the pathophysiologic mechanisms and neuronal network alterations that underlie TBI [6]. The fluid-percussion injury (FPI) model for closed-head injury is one of the most widely used animal models of TBI [7,8]. Several studies using the lateral FPI model report widespread axonal injury following severe TBI [9,10,11], such as in white matter structures, including the internal capsule and corpus callosum [12]. Post-TBI axonal injury affects major cortical and subcortical networks, such as the cortico-thalamo-cortical network.

Substantial evidence reveals alterations in the thalamo-cortical network following TBI in clinical and preclinical models of TBI [13,14,15]. The cortico-thalamo-cortical network plays a critical role in the flow of information between the thalamus and sensory areas of the cortex. Axons from different thalamic nuclei target specific cortical areas and layers [16,17]. The epicenter of the post-TBI cortical lesion induced by the lateral FPI model is usually located in the auditory and sensory cortices [18]. Injury to the thalamus is also not uncommon in this model [8,12,19]. Injury to the auditory and sensory cortices can severely affect their connection to the ventral and dorsal medial geniculate, and the ventral posterior lateral (VPL) and medial (VPM) thalamic nuclei, respectively, which receive their inputs [20]. A widely investigated cortico-thalamo-cortical network is the connection between the somatosensory cortex and the VPL and VPM thalamic nuclei [21]. Axons from the VPL and VPM target layers IV and VI of both the primary somatosensory cortex (S1) and secondary somatosensory cortex (S2) [16,21]. Several preclinical studies have shown extensive neurodegeneration in the thalamic VPL and VPM. Indeed, the degeneration of the thalamic relay neurons from the VPL and VPM results in pathology in the primary somatosensory cortex barrel field (S1BF) [22,23]. How TBI affects the reorganization of this network and how the post-injury reorganized network affects the functional outcome, however, remain unclear.

In the present study, we investigated the structural reorganization of VPL/VPM axonal projection terminals in S1 and the functional activity of the VPL/VPM thalamo-cortical network following TBI in rats injured using the lateral FPI model. Considering the well-documented damage to the somatosensory cortex and thalamus after lateral FPI, we hypothesized that TBI causes a reduction in the density of the VPM/VPL thalamo-cortical axonal projection terminals and leads to changes in their orientation within the layers of S1, as well as inclines the network activity towards a state of hyperexcitability. Our findings indicated that TBI did not alter the laminar specificity of the axonal terminals in S1, but did cause a reduction in their density as well as loss of the dense plexus formation in layer IV and VI observed in sham-injured controls. Furthermore, TBI altered the orientation of the axonal terminals from a perpendicular alignment with the pial surface to a more parallel alignment in layer V. Functional analysis revealed that the structurally altered VPL/VPM thalamo-cortical network show signs of hyperexcitability, observed as an increased beta activity following optogenetic stimulation.

## 2. Results

### 2.1. Mortality

Out of 11 TBI rats, three died acutely (27% post-impact mortality) and none died during the follow-up period. The mean impact pressure was 3.19 ± 0.02 atm (mean ± SEM) and the mean apnea duration was 27 ± 5 s. Impact-induced seizure-like behavior, which included tail rotation, jerks, and movement of the lower torso, was observed in 36% (4/11) of the rats. The surviving rats (*n* = 8), including shams (two controls), were injected at 1-month post-injury with the viral vector carrying ChR2 opsin with eYFP as the reporter (rAAV5/CaMKII-hChR2(H134R)-eYFP-WPRE).

### 2.2. ChR2-eYFP Opsin Traced VPL/VPM Axonal Projections to S1

To assess whether lateral FPI-induced injury in the cortex and thalamus was associated with structural reorganization of the VPL/VPM projections to the perilesional cortex, we first examined the organization of the thalamocortical projections to S1 from immunohistochemically stained brain sections. Broad histologic examination revealed strong ChR2-eYFP expression in the thalamus and cortex in both controls and four TBI rats. We eliminated three of the other four TBI rats that exhibited very low or negligible ChR2-eYFP fluorescence in the thalamus and cortex suggesting an unsuccessful probe injection in the thalamus and/or tracer transport to the cortex. One with moderate expression was included in the study (see Supplementary Figure S1). Interestingly, these cases with very low or moderate ChR2-eYFP expression showed no EEG activity upon optogenetic stimulation (data not shown). Consequently, only the animals that showed strong or moderate tracer fluorescence in the thalamus and cortex were included in the structural analysis (five TBI, two controls). Those with a strong expression of ChR2-eYFP and EEG activity upon optogenetic stimulation were included in the functional analysis (four TBI and two controls).

#### 2.2.1. Location of the Viral Injection and Opsin Expression in the Thalamus

**Figure 1:**
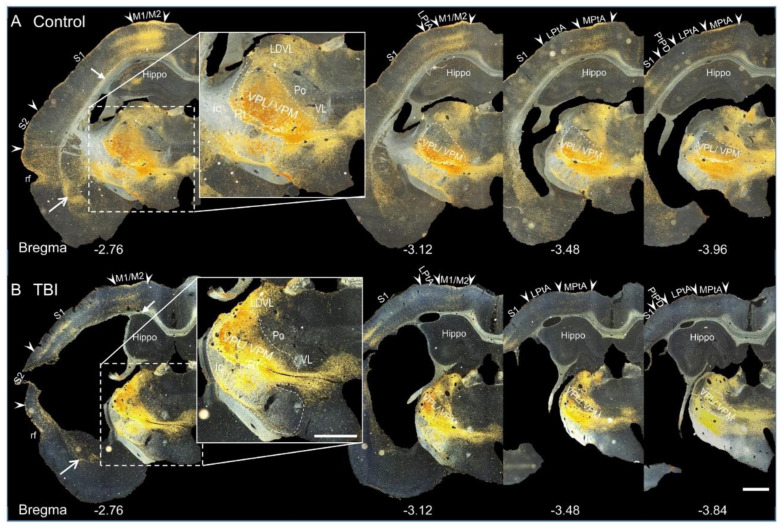
Darkfield photomicrographs demonstrating the distribution of ChR2-eYFP expression in CAMKII neurons (CaMKII-eYFP) in the thalamus of a control and traumatic brain injured (TBI) rat at 3 months after viral transduction. (**A**) ChR2-eYFP expression in a sham-injured (control) rat. ChR2-eYFP expression is revealed here as eYFP-positive immunolabeling (golden yellow color). ChR2-eYFP expression is observed in the thalamus, cortex, amygdala (open arrow), internal capsule (ic), external capsule (ec), and corpus callosum (cc) (closed arrow). The most intense ChR2-eYFP expression is in the targeted VPM and VPL thalamic nuclei. Note the ChR2-eYFP expression in the adjacent thalamic nuclei (insert), such as the laterodorsal thalamic nucleus (LDVL), post thalamic nuclear group (Po), and ventral thalamic nucleus (VL). (**B**) Darkfield image of ChR2-eYFP expression in a TBI animal. The thalamic expression pattern is comparable to that in controls (compare with panel A). Abbreviations: Hippo, hippocampus, ic, internal capsule; LPtA, lateral parietal association cortex; M1, primary motor cortex; M2, secondary motor cortex; Po, posterior thalamic nuclear group; Rt, reticular nucleus; S1, primary somatosensory cortex; S2, secondary somatosensory cortex; VPL, ventral posteromedial thalamic nucleus; VPM, ventral posterolateral thalamic nucleus. Scale bars in A, B, and the insert equals 1 mm.

**Figure 2:**
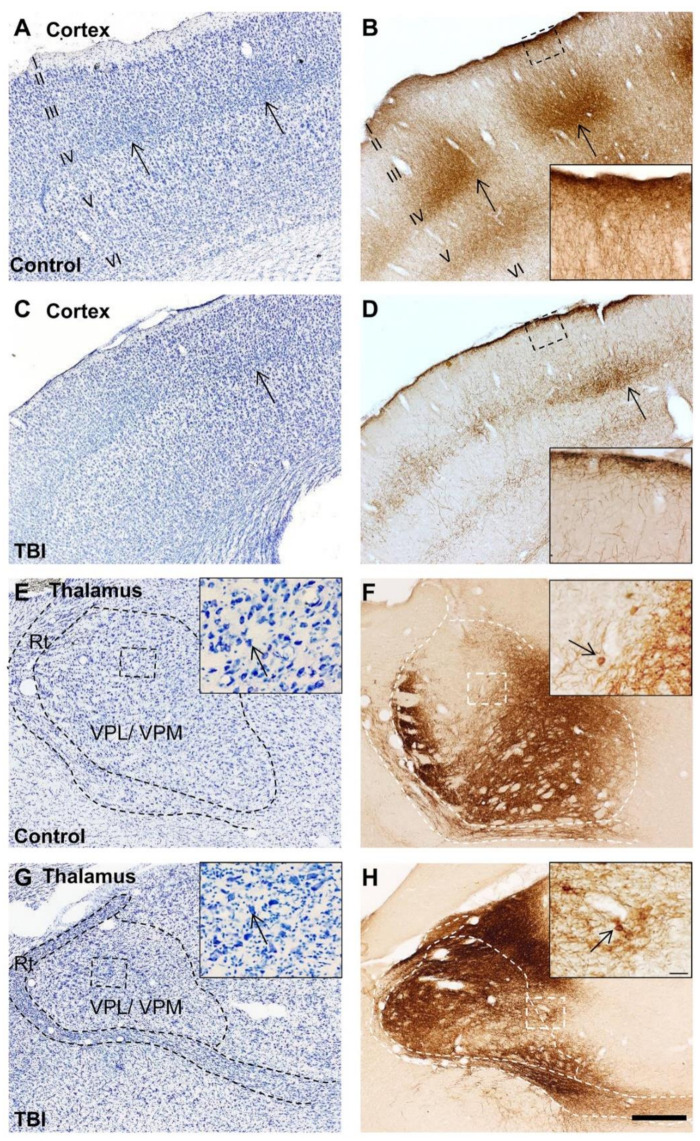
Brightfield photomicrographs of the cortex and thalamus of thionin-stained (**left panel**) and the adjacent immunostained eYFP-labeled (**right panel**) sections of a control and traumatic brain injured (TBI) rat. (**A**) A coronal section of the cortex of a control rat stained with thionin, ipsilateral to the viral injection. (**B**) The adjacent section of the control rat in (**A**) demonstrating eYFP-immunoreactivity (ir) (ChR2-eYFP expression) in the cortex. A dense plexus of ChR2-eYFP-expressing varicose fibers (axons) innervates cellular clusters in layers IV and V (open arrow). Note that immunopositive axons send collaterals into layer I (see insert). (**C**) A thionin-stained coronal section of the perilesional cortex from a rat with TBI. Note the similar group of cells as in the control rat (open arrow). (**D**) An adjacent section of the TBI rat in panel C, demonstrating ChR2-eYFP-expressing varicose fibers in the cortex. Note the decreased axonal density and less clear columnar organization of immunopositive terminals in layers IV and V. Also, the density of immunopositive terminals in layers II and III was lighter and the terminal plexus was more scattered in the TBI rat (insert). (**E**) A thionin-stained section showing the ipsilateral thalamus of a sham-operated experimental control rat. There was no cell loss in the ventral posterolateral (VPL) and ventral posteromedial (VPM) thalamic nuclei (see insert). (**F**) ChR2-eYFP expression in the thalamus from the section adjacent to panel E. Note the expression of ChR2-eYFP in the cells of the VPL and VPM nuclei (insert). (**G**) A thionin-stained section of the ipsilateral thalamus of a TBI rat. Note the atrophy and neurodegeneration in the VPL and VPM thalamic nuclei. The remaining neurons of the VPL and VPM express ChR2-eYFP (insert). (**H**) The adjacent section to panel G, showing ChR2-eYFP expression in the VPL and VPM thalamic nuclei. Note the neuronal expression of ChR2-eYFP (insert). Scale bar in A–H equals 400 µm and in the insert equals 40 µm. Abbreviation: Rt, reticular nucleus.

The VPL and VPM thalamic nuclei were the targets of the viral injection. At 3 months after the injection, sham-injured (controls) and TBI rats showed strong expression of ChR2-eYFP in the ipsilateral thalamus (Figure 1). The ChR2-eYFP expression in the thalamus was more pronounced in the ventral aspects than in dorsal aspects and spread caudally (Figure 1 and Figure 2). Expression of ChR2-eYFP was particularly strong in the transduced cells of the VPM and VPL nuclei (Figure 1 and insert in Figure 2F,H). ChR2-eYFP-expressing fibers were also observed in other thalamic nuclei adjacent to the VPL/VPM, including the post thalamic nuclear group (Po) and the ventrolateral (VL) and ventromedial (VM) thalamic nuclei (Figure 1). Moreover, we also observed ChR2-eYFP-expressing fibers in the laterodorsal thalamic nucleus and amygdala (Figure 1 open head arrows). In all cases, ChR2-eYFP-expressing varicose-appearing fibers were observed in white matter structures such as the internal capsule and the corpus callosum (Figure 1 closed head arrows). ChR2-eYFP-expressing fibers were rarely observed in distant areas such as the hypothalamus or contralateral thalamus (Figure 1 and Figure 2). The thalamic expression pattern was similar between controls and TBI animals (Figure 1).

#### 2.2.2. ChR2-eYFP Expression in the Cortex after TBI

**Figure 3:**
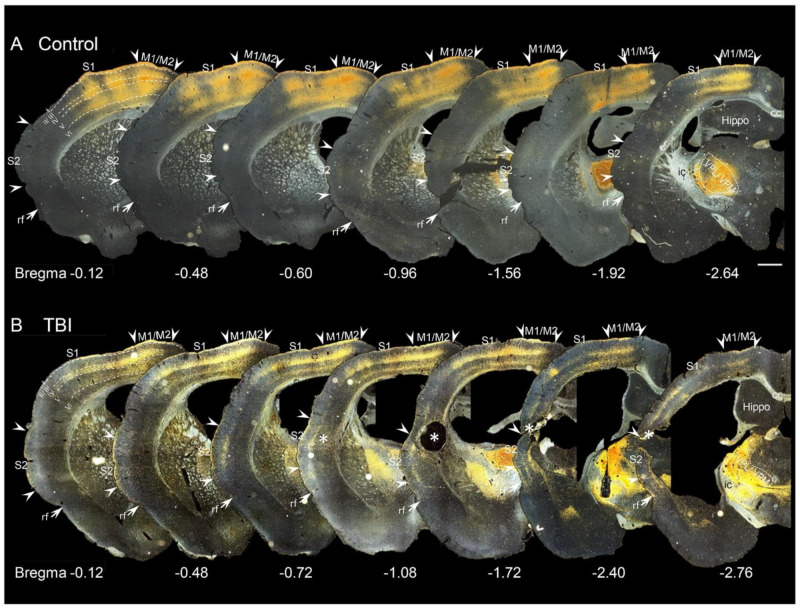
Coronal darkfield photomicrographs demonstrating the anteroposterior distribution of GFP-labeled ChR2-eYFP-expressing axonal terminals in the cortex of a control and traumatic brain injury (TBI) rat. (**A**) ChR2-eYFP expression in the cortex of a control rat. The expression ChR2-eYFP in the cortex extended anteriorly with intense expression noticeable in the primary (M1) and secondary (M2) motor cortices as well as in the primary somatosensory cortex (S1). Few ChR2-eYFP-expressing fibers are observed in the secondary somatosensory cortex (S2). (**B**) ChR2-eYFP expression in the cortex of a TBI rat. The pattern of ChR2-eYFP expression is similar to that in the control in panel A. Note the decrease in fiber density and the absence of columnar fiber bundles compared with that in a control rat (panel A). ChR2-eYFP-expressing varicose fibers were also observed around the lesion core (asterisk). Scale bar equals 1 mm. Abbreviations: Hippo, hippocampus; ic, internal capsule; rf, rhinal fissure; VPL, ventral posterolateral thalamic nucleus; VPM, ventral posteromedial thalamic nucleus.

At 3 months post-TBI, expression of ChR2-eYFP in the axonal terminals in both controls and TBI rats was observed within ipsilateral M1 and M2, and S1 and S2. ChR2-eYFP expression was stronger in S1 and M1/ M2 compared with S2 (Figure 2B,D and Figure 3). In no case were there ChR2-eYFP-expressing fibers in the cingulate cortex, lateral cortical areas such as the piriform cortex, or in the contralateral cortex.

In the cortex, ChR2-eYFP-expressing fibers spread more anteriorly than caudally from the level of the injection site (Figure 3). In the cortical layers, the fibers formed a dense plexus in layers IV and VI (at the border between layers V and VI) (Figure 2B,D and Figure 3). These dense plexuses were sometimes observed to occur in columns, about 300 µm wide and 500 µm apart (Figure 2 and Figure 3). This organization was particularly evident in controls and conspicuously absent in TBI animals (Figure 2B,D). Terminal collaterals extended from layer IV through layers II and III into layer I (insert in Figure 2B,D). In layers II and III, the collaterals ran perpendicular to the pial surface. In layer I, the collaterals ran parallel to the pial surface in both controls and TBI rats, but it was particularly conspicuous in controls (Figure 2B,D, see also Figure 3).

### 2.3. PHA-L Tracer Confirmed ChR2-eYFP-Traced VPL/VPM Thalamocortical Tracks

To confirm the finding that axons of ChR2-eYFP-transfected VPL/VMP neurons projected to S1, we analyzed immunolabeled PHA-L brain sections of naïve rats injected with the anterograde tracer PHA-L into the VPL/VMP thalamic nucleus of the right hemisphere.

#### 2.3.1. Location of Tracer Injection

**Figure 4:**
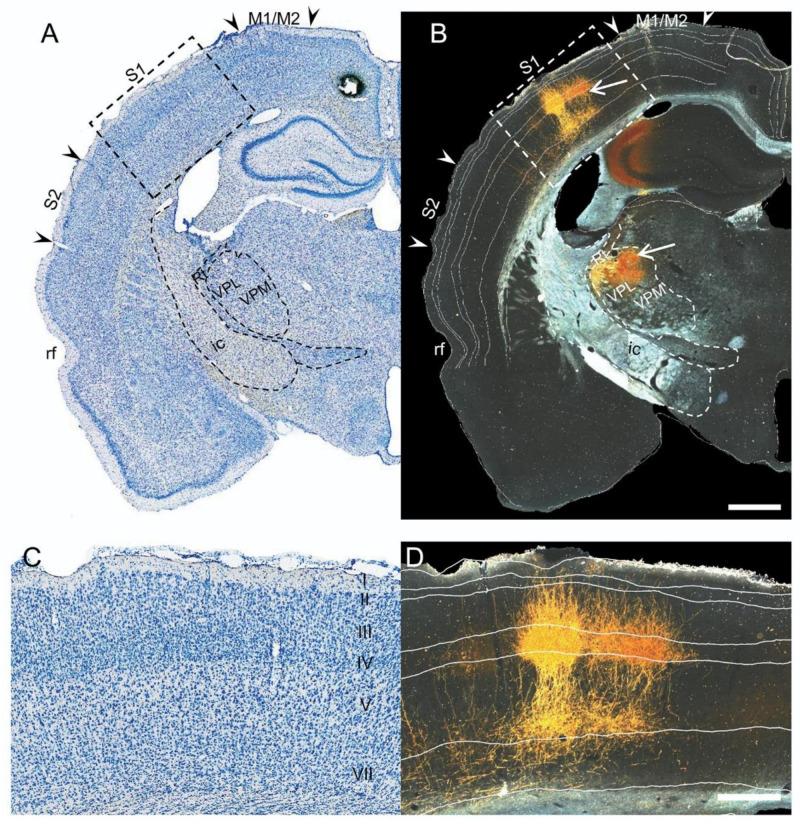
Brightfield and darkfield photomicrographs of a thionin-stained (**left**) and PHA-L immunolabeled (**right**) sections of a naïve rat. (**A**) Thionin-stained section of a left hemisphere ipsilateral to thalamic PHA-L injection. The PHA-L injection was targeted to the thalamic ventral posterolateral (VPL) and ventral posteromedial (VPM) nuclei. (**B**) A darkfield image of the rat left hemisphere with PHA-L immunoreactivity (ir). PHA-L-ir in the thalamus is observed in the VPL and VPM thalamic nuclei (open arrow). The efferent terminals from the VPL and VPM innervated the primary somatosensory cortex. (**C**) High magnification of the area within the dashed line in panel A depicting the cortical layers. (**D**) High magnification of the area within the dashed line in panel B, depicting the laminar distribution of the axonal terminals. Note the dense axonal plexus in layer IV and the border between layers V and VI. Collaterals traveled through layers II/ III into layer I. Scale bar in A and B equals 1 mm, and in C and D equals 400 µm.

Immunohistochemical labeling of brain sections revealed PHA-L immunoreactivity (PHA-L–ir) in the thalamus and cortex as in the ChR2-eYFP cases (Figure 4B). In the thalamus, the labeling was mainly observed in the target VPL/VPM thalamic nuclei (Figure 4B). The tracer was not observed in other thalamic nuclei as with the ChR2-eYFP tracer, except for cases in which the targeted injection site was missed (data not shown). Additionally, the PHA-L tracer was exclusively located in the ipsilateral thalamus.

#### 2.3.2. Cortical Expression of the PHA-L Tracer

In the cortex, the PHA-L-labeled terminals were exclusively observed in ipsilateral S1 (Figure 4). As with the ChR2-eYFP tracer, the axons formed a columnar dense plexus in layers IV and VI (between the border of layers V and VI), with collaterals extending perpendicularly into layers III and II. The tips of some terminals were observed in layer I. There were no collaterals running parallel to the pial surface within layer I, as observed with the ChR2-eYFP tracer (Figure 4B). Furthermore, as with ChR2-eYFP, PHA-L–ir terminals extended rostrally rather than caudally from the level of the injection site.

### 2.4. Changes in the Density of ChR2-eYFP-Expressing Fibers in the Cortex after TBI

**Figure 5:**
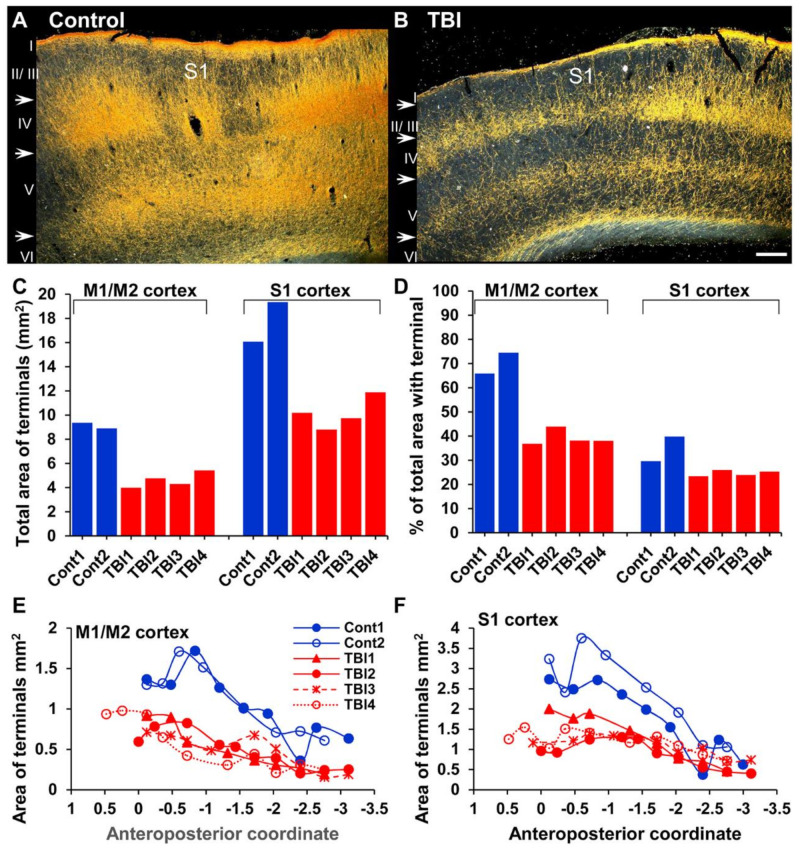
Rostrocaudal distribution of the density of ChR2-eYFP-expressing fibers in the motor and primary somatosensory (S1) cortex of control and traumatic brain injured (TBI) rats. (**A**) Darkfield image of the S1 cortex of a control rat. Note the high density of ChR2-eYFP-expressing fibers. (**B**) Darkfield image of S1 of a TBI rat. Note the reduced density of the ChR2-eYFP-expressing fibers. (**C**) Bar graph showing the total area of the ChR2-eYFP-expressing fibers in the primary (M1) and secondary (M2) motor cortices, and S1. There was a high fiber density in controls as compared to TBI rats in both the M1/M2 and S1 cortices. (**D**) Bar graph showing the percentage of M1/M2 and S1 covered by ChR2-eYFP expressing fibers. (**E**) Rostrocaudal distribution of the density of ChR2-eYFP-expressing fibers in the motor cortex. Note the high density of fibers in rostral sections in both controls and TBI rats. (**F**) Rostrocaudal distribution of ChR2-eYFP-expressing fibers in S1. The density of ChR2-eYFP-expressing fibers is higher in rostral sections compared with the caudal sections. Scale bar equals 200 µm.

The laminar distribution of projection terminals in the cortex did not differ between TBI and control rats, but the density of the projection terminals within the different cortical layers seemed to vary. Visual inspection of the ChR2-eYFP–ir sections indicated that the density of ChR2-eYFP-expressing fibers was high in M1/M2 and S1 in controls and TBI rats. Some ChR2-eYFP-expressing fiber terminals were also observed in S2 (Figure 1 and Figure 3). Quantitative analysis confirmed that the average total area of ChR2-eYFP-expressing fibers in M1/M2 was 9.13 mm^2^ (70.2% of total area of ROI, *n* = 2) in controls and 4.63 mm^2^ (39.2% of total area of ROI, *n* = 4) in TBI rats (Figure 5). In S1, the total area of ChR2-eYFP-expressing fibers was 17.7 mm^2^ (34.8% of total area of ROI, *n* = 2) and in controls 10.2 mm^2^ (24.7% of total area of ROI, *n* = 4) in TBI rats (Figure 5). In controls and TBI rats, the density of the ChR2-eYFP-expressing fibers in M1/M2 and S1 decreased rostrocaudally (Figure 5E,F). Statistical comparison between TBI and Sham rats transduced with ChR2-eYFP was not possible since only two shams were included in the study.

### 2.5. Changes in the Orientation and Anisotropy of ChR2-eYFP-Expressing Fibers in the Cortex after TBI

To investigate whether TBI affected the cortical organization of thalamocortical fibers, we further assessed the anisotropy and orientation dynamics of the ChR2-eYFP and PHAL-expressing fibers in cortical layer V of the S1 within a selected area (~2 to 3 mm from the medial edge of the lesion in TBI cases). This area, described as the perilesional cortex, was previously reported to be epileptogenic [24,25].

**Figure 6:**
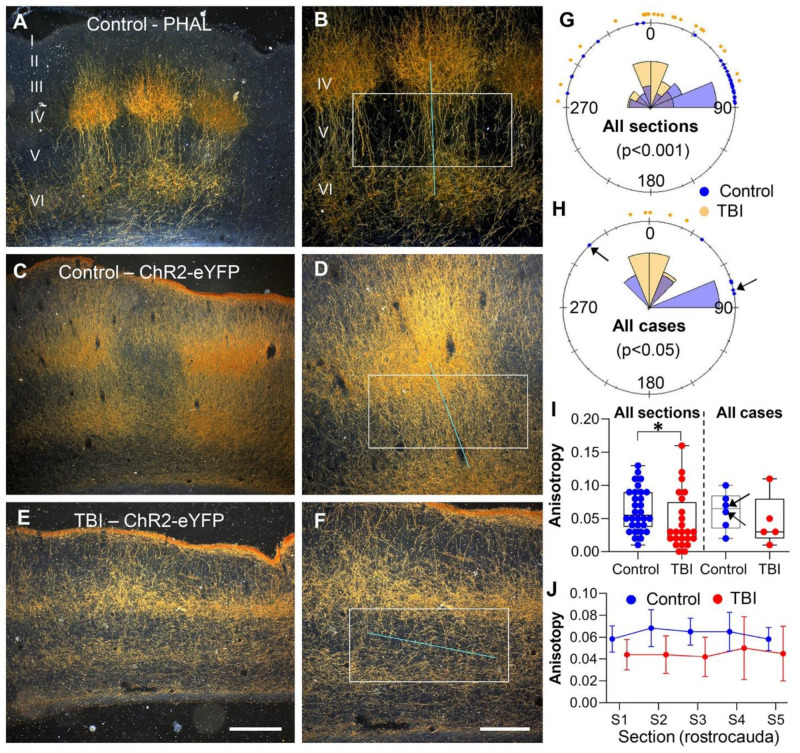
Orientation and anisotropy of ChR2-eYFP and PHAL-expressing fibers in layer V of the primary somatosensory (S1) cortex of a ChR2-eYFP sham-operated, PHAL-näive, and traumatic brain injury (TBI) rat. (**A**) Darkfield photomicrograph of the S1 demonstrating PHAL-ir thalamo-cortical terminals in the S1 cortex of a naïve rat. (**B**) A high-magnification photomicrograph from panel (**A**) demonstrating the average directionality of the immunopositive fibers (blue line) and anisotropy (length of the blue line) within the selected region of interest (ROI) (white selection). The spatial orientation and anisotropy of the fibers was assessed using the “FibrilTool” in ImageJ^®^. The orientation is estimated based on the x-axis of the image. All the cortical images were aligned in the same way for the analysis. Note the almost 90° orientation and high anisotropy of the fibers (ordered and parallel) in layer V. (**C**) ChR2-eYFP-expressing fibers in the S1 of a sham-operated rat. (**D**) A high-magnification photomicrograph from panel (**C**). Note the perpendicular orientation of the fibers with respect to the pial surface is comparable to that in PHAL-naïve rats. (**E**) ChR2-eYFP expressing fibers in S1 of a TBI rat. Note the absence of fiber bundles seen in controls (panels **A** and **C**). (**F**) A high magnification of panel (**E**) demonstrating a parallel orientation of the fibers in layer V within the selected ROI. (**G**) A graphical representation of the orientation in all the sections of control and TBI rats analyzed. The fibers in the controls were orientated almost perpendicular (90°) to the pial surface in comparison to TBI rats (Watson’s two-sample test). (**H**) A graphical representation of the average orientation of fibers in each rat (Watson’s two-sample test). (**I**) A box and scatter plot demonstrating the anisotropy of the fiber in layer V of each section analyzed and the mean value in each control and TBI rat. The high anisotropy values in controls (Mann-Whitney U test) demonstrates more parallel and ordered fibers as compared to that in TBI rats. (**J**) Rostrocaudal distribution of the anisotropy in control and TBI rats. Data are presented as mean ± SEM. Arrows in H and I indicate ChR2-eYFP sham control rats. Scale bar in panels **A**, **C,** and **E** equals 400 µm and in panels **B**, **D,** and **F** equals 200 µm. * *p* < 0.05.

Visual inspection of the brain sections revealed that the eYFP or PHAL expressing fibers in layer V were orientated perpendicular to the pial surface in sham and PHAL-naive controls (Figure 6A–D). In TBI rats (ChR2-eYFP only), the fibers were orientated parallel to the pial surface (Figure 6E,F). Rostrocaudal analysis of the fiber orientation using the FibrilTool plugin in ImageJ software revealed that the mean fiber orientation relative to the pial surface in the sham-operated ChR2-eYFP-expressing controls (*n* = 2) did not differ from that in naïve PHAL-injected controls (*n* = 4). Consequently, data from these animals were pooled together for further analysis. The mean orientation of the fibers differed between the controls (*n* = 6, 60.30°) and TBI rats (*n* = 5, 5.43°, *p <* 0.05, Figure 6G and H) (five sections/animal, bregma level +0.4 to −2.4, 300 µm apart). Similarly, the mean anisotropy in all the sections analyzed (bregma level +0.4 to −2.4, 300 µm apart) differed between controls and TBI animals (0.063 ± 0.006 vs. 0.045 ± 0.009, *p <* 0.5, Figure 6I). However, there was no difference between controls and TBI rats in the mean anisotropy (*p >* 0.05, Mann-Whitney U test) (Figure 6I) or the rostrocaudal distribution of anisotropy (Figure 6J).

### 2.6. Increased Beta Activity in TBI Rats following Optical Stimulation

To assess whether the altered density and orientation of the thalamocortical pathway in TBI animals modulated the activity of thalamo-cortical networks, we probed the functional output of the thalamic-cortical projections from the VPL/VPM to perilesional S1 using optogenetics.

Analysis of the baseline EEG recordings revealed no spontaneous epileptiform activity during the 1-week follow-up period. Age-related spike-wave-discharges were observed in one control and five TBI rats [26].

**Figure 7:**
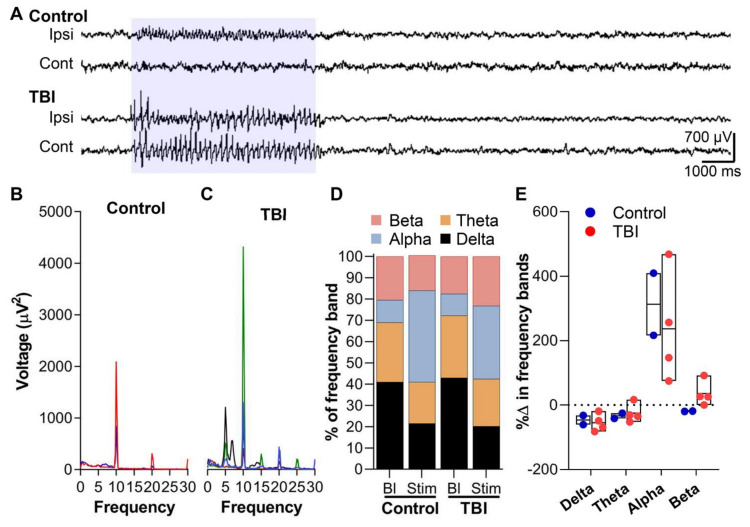
Optogenetic activation of the channel rhodopsin 2 (ChR2)-transduced thalamo-cortical network. (**A**) EEG recordings demonstrating spiking unilaterally in the control and bilaterally in the TBI rat during a 5-s 10 Hz optogenetic stimulation with blue light (470 nm). Note the high-voltage activity in the TBI rat compared with controls. (**B**) Spectral analysis of EEG activity during optical stimulation in control rats showed peak activity at 10 Hz stimulation frequency. Also, note the other peaks at 20 and 30 Hz. (**C**) TBI rats with spiking activity in the EEG also displayed a peak activity at 10 Hz in the EEG spectral analysis during the optical stimulation. Unlike the controls, however, they showed repeated peaks at every 5-Hz interval. (**D**) The percentage of the voltage of the different frequency bands at baseline and during the optical stimulations in controls and TBI rats. (**E**) Suspended box plot of percentage change in voltage of the frequency bands normalized to baseline values. Control and TBI rats showed an increased alpha frequency (corresponding to the stimulation frequency) whereas only TBI rats showed a tendency towards a high beta activity, indicative of possible network hyperexcitability.

When the rats were photostimulated, all controls (*n* = 2) and four of eight TBI rats showed a cortical response in the EEG. The photostimulation produced sharp spikes in the ipsilateral S1 cortical electrode lasting for the duration of a 5-s stimulation. In TBI rats, however, the similar photostimulation resulted in bilateral fast-spiking activity (Figure 7A). Spectral analysis of the ipsilateral cortical response showed prominent high voltage 10-Hz activity in both the control and TBI rats (Figure 7B,C). In controls, the mean peak voltage of the 10-Hz activity was 1461 uV^2^ (range: 834–2087 uV^2^) (Figure 7B). In the TBI animals, the peak voltage was 1733.3 uV^2^ (range: 406–4314 uV^2^) (Figure 7C). Furthermore, controls showed other peak frequencies at 10-Hz intervals (20 Hz and 30 Hz), whereas TBI rats showed frequency peaks at 5-Hz intervals (Figure 7C). A spectral analysis of the different frequency bands during the stimulation, normalized to baseline values, revealed that both the sham and TBI rats showed decreased delta and theta activity but increased alpha activity (Sham: 312%, TBI: 237%) (*p >* 0.05, χ^2^ test) (Figure 7D). However, only the TBI rats showed increased beta activity (133%) (*p >* 0.05, χ^2^ test) during the optical stimulation (Figure 7D). Despite the fact that only a subset of neurons are being optically activated, their response was suggestive of a hyperexcitability state.

## 3. Discussion

Axonal injury, a hallmark of severe traumatic injury, disrupts cortical and subcortical networks and may lead to functional deficits and poor clinical outcomes [3,5,9,10]. Elucidating the details of post-TBI cortical network reorganization is crucial toward understanding the plastic changes that underlie the development of functional deficits and outcomes. We hypothesized that TBI induced by lateral FPI causes changes in the density, orientation, and terminal targets of projections from the thalamus to the S1 perilesional cortex, an area previously reported to be epileptogenic [24,25]. Consequently, the thalamo-cortical network activity is changed, particularly in the target areas of the VPL/VPM projections. Our data demonstrate that after TBI, (1) the density of the projections terminals in S1 is reduced, (2) the laminar-specificity of the thalamo-cortical projections from the thalamic VPL and VPM is not altered, (3) the orientation of the terminals in layer V with respect to the pial surface changes from a perpendicular alignment in controls to a parallel alignment in TBI animals, and (4) the modulated thalamo-cortical network activity in response to optogenetic thalamic stimulation reflects hyperexcitability.

### 3.1. The Overall Spatial Distribution of Thalamo-Cortical Axonal Terminals in the Cortex Is Not Altered by TBI

To investigate whether TBI causes structural changes in the organization of thalamocortical axonal terminals, we examined the spatial and laminar patterns of ChR2-eYFP fiber terminals in the perilesional cortex. We used eYFP as a reporter for ChR2 opsin expression in CaMKII neurons. ChR2-eYFP-expressing fibers were observed in the thalamus and cortex in both controls and TBI rats, indicating that the viral transduction was successful, irrespective of the injury. In the thalamus, ChR2-eYFP expression was observed in the cell bodies and axonal fibers, as previously described [27]. The highest density of ChR2-eYFP fibers was seen in the targeted VPL/VPM nuclei but also in other thalamic nuclei, especially the dorsal aspect of the thalamus, such as the laterodorsal thalamic nucleus. The expression in other thalamic nuclei might be due to either backflow during retraction of the injection needle or poor targeting of the injection site. Interestingly, we also observed ChR2-eYFP expression in the amygdala, particularly in the basal nucleus, suggesting sensory input from the VPL/ VMP. In fact, an elegant study by Turner and Herkenhan showed sensory input from the thalamic ventral posterior nucleus and paratenial nucleus to the lateral and basolateral nuclei of the amygdala using ^3^H-amino acid autoradiographic tract-tracing [28]. Also, the thalamic ventral posterior nucleus projects to the dorsal division of the lateral nucleus of the amygdala (for a review, see [29]).

From the injection site in the thalamus, ChR2-eYFP fibers also extended through the external capsule into the internal capsule and corpus callosum, and then turned tangentially into the cortex and terminated in S1/S2 and M1/M2. The sensory thalamic nuclei, VPM/VPL, project to the somatosensory cortex [30,31,32]. The observation of projection terminals in the motor cortex, however, suggests that neurons of the motor thalamic areas, the VL and VA complex, were most likely transduced as well. Our data confirm previous findings by Kuramoto and colleagues, who used single-neuron tracing following viral vector transduction to show that neurons in the VL–VA complex project to M1/M2 and S1 [17].

Thus, our data indicate that axonal fibers from the VPL and VPM nuclei project to S1 and send sensory inputs into the amygdala. The overall spatial distribution of VPL/VPM axonal efferents is not altered following TBI.

### 3.2. TBI Does Not Alter the Laminar-Specificity of Thalamo-Cortical Axonal Terminals, but Rather Changes Their Orientation

Thalmo-cortical axonal projections from the thalamic VPL and VPM nuclei to the somatosensory cortex are extensively described [20]. The present findings are consistent with previous reports demonstrating that axonal projections from the thalamic VPL and VPM nuclei target the somatosensory cortex, with the projection terminals targeting principally layer IV and the border between layers V and VI, described in some studies as layer VI [31]. This was also confirmed by the PHA-L injection into the VPL/VPM nuclei of naïve rats. The anatomical targets were comparable between naïve PHAL and ChR2-eYFP-sham rats, thus we did not consider PHAL injection for TBI rats. However, we observed no projection terminals in layer I with the PHA-L, as was the case with ChR2-eYFP transduction. This may be due to the short duration (10 days) allowed for PHA-L uptake and transportation, whereas the expression of ChR2-eYFP went on for several weeks. PHA-L is transported at a speed of 4 to 6 mm per day [33]. The axonal length of the VPM neurons that project to S1 is estimated to be 86,968.8 µm [31]. This suggests that approximately 14 days is needed to completely label the VPL/VPM neurons, including all their terminals and the layer I collaterals. However, the terminals in layer V, our area of interest, were already labeled within 10 days as demonstrated in the results. Axonal projection collaterals to layer I were demonstrated in earlier studies. For example, Oda and colleagues (2004) injected a biocytin tracer either extracellularly or intracellularly into the VPM of Sprague Dawley rats to show that projections originating from the VPM form a dense plexus in layers IV and VI and project collaterals to layer I of S1 [31].

In the present study, TBI did not alter where the thalamo-cortical projections terminated in the cortex as discussed above. Rather, it was very clear that the bundles of dense plexuses, which were obvious in controls, were conspicuously absent in the injured animals. This finding confirms previous findings showing a similar loss of axonal terminal organization in the S1BF following diffuse brain injury by the midline FPI [22,23]. These dense plexuses, which were typically located in the S1BF, contact the barrel cells [34]. Our data indicate that TBI may compromise the whisker somatosensory circuit. There is some evidence that whisker sensitivity is impaired following diffuse brain injury induced by the midline FPI [23,35,36]. Further evidence in the lateral FPI model indicates hypoactivity within the ipsilateral barrel cortex and chronically depressed whisker stimulus-evoked local cerebral metabolic rates of glucose in the somatosensory cortex [37,38]. These data suggest compromised whisker circuitry.

A very interesting observation was that the orientation of ChR2-eYFP-expressing axonal terminals in the cortex appeared more diffuse or scattered in TBI rats, particularly in layers II to V. Also, in TBI rats, the peculiar vertical orientation of the fibers in layer VI traveling toward layer V observed in controls was lost. The abnormal orientation of the axonal terminals suggests possible reorganization of the surviving thalamocortical projection terminals. Several studies provide evidence for structural and functional changes in the somatosensory cortex following TBI, suggesting ongoing dynamic reorganization. For example, we previously showed neurodegeneration in the somatosensory cortex following experimental TBI and reported that the severity of the cortical damage was associated with increased hyperexcitability induced by pentylenetetrazol (discussed below) [18,39,40]. Evidence from human TBI diffusion tensor imaging studies shows low fractional anisotropy (FA) and a high mean diffusivity/apparent diffusion coefficient (MD/ADC) in several brain areas including the cortex, following severe brain injury, indicative of white matter damage (axonal damage) [41]. Diffusion tensor imaging-tractography studies have also revealed widespread changes in white matter tracts in TBI patients, where widespread or reduced tracts are reported to be associated with low Glasgow coma scale scores [42]. An elegant preclinical study by Salo and colleagues showed that in the deeper layers of ipsilateral S1 in rats injured by lateral FPI and killed 5 months after injury, the FA values were decreased, and the orientation of the water diffusion was changed from a rostrocaudal direction in shams to multiple directions in TBI rats. These findings suggest a possible scattered orientation of the axonal terminals following TBI [43]. Johnstone and colleagues also reported similar changes in FA and tract density in the ipsilateral barrel cortex in the lateral FPI model [44]. Our tract tracing study extends findings from previous studies by specifying the structural reorganization of the thalamo-cortical terminals as a component of S1 post-injury diffusion changes.

Taken together, our data indicate that TBI does not alter the laminar-specific targeting of the axonal projections from the VPM thalamic nucleus in the somatosensory cortex. The dense plexus of the thalamo-cortical axonal terminals in cortical layer IV is completely lost and the orientation of the axonal terminals in the superficial layers, except in layer I, appear scattered, whereas those in the deeper layer VI appear to run parallel to the pial surface.

### 3.3. The Density of the Thalamo-Cortical Projections in the Cortex Was Reduced Following TBI

To further characterize the TBI-related structural reorganization of the thalamo-cortical axonal terminals, we examined whether the reduced density of axonal terminals following TBI, particularly within the ipsilateral thalamus, altered the density of the terminals within the perilesional cortex.

The expression of ChR2-eYFP in the axons and axonal terminals depends on the success of the viral transduction of the transgene. Stronger expression was noted in all controls compared with TBI rats. Four of eight TBI rats showed little or no ChR2-eYFP expression in the cortex or thalamus and these cases, not surprisingly, displayed no EEG activity upon optical stimulation. There are several possible reasons for the lack of expression in these cases. Reports suggest that the volume of the viral load is inversely related to the rate of transduction of a transgene [45,46]. In this study, all the rats received the same viral load volumes, suggesting that other factors are responsible for the low transduction rate in the four TBI rats. Ongoing secondary injury processes in the thalamus after TBI are another possibility [47,48,49]. TBI induces a host of innate and adaptive immune responses that can be hostile against the viral particles and their gene products, thus decreasing the effectiveness of the transduction [47,48,49,50]. Although adeno-associated viruses are known to induce very little immunogenicity in murine models, we avoided any possible complication from the acute post-TBI inflammatory process by injecting the virus 1 month after the injury when the injury-induced acute inflammation was significantly reduced [51,52]. Further investigations are needed to determine whether the transduction success or lack thereof was influenced by the post-injury time-point of viral injection. Furthermore, the ensuing chronic inflammation post-TBI, which proceeded several months after the initial injury, can also affect the transduction. To the best of our knowledge, this is the first study to use opsin-based anterograde tracers injected into the thalamus at the chronic phase post-TBI to track the thalamo-cortical network. Furthermore, the success of the transduction of thalamic CaMKII neurons in the VPL/VPM at 1 month after TBI also to a large extent depends on the number of neurons surviving after the injury. It is worth recalling that four TBI rats had a low to moderate expression of ChR2-eYFP in the thalamus and the cortex. Several issues may have contributed to this including the number of cells transduced with the tracer, pathology-related issues regarding axonal expression or transportation of tracer, inaccurate targeting due to variability in bregma reading, or TBI associated rotation of the brain and atrophy. However, we did not observe a huge ChR2-eYFP expression in other thalamic nuclei or cortical targets suggesting a missed target. On the other hand, chronic neurodegeneration as described in several studies and confirmed here, may have likely affected the transduction of the transgene in the VPL/VPM nucleus [12,19]. Although we did not quantify the severity of thalamic neurodegeneration in the individual TBI cases, our data indicate that the severity of the neurodegeneration may have influenced the level of ChR2-eYFP expression in the TBI cases.

In the TBI cases with successful expression, the density of the ChR2-eYFP-expressing fibers in the cortex was decreased compared with that in controls. In fact, the density of the ChR2-eYFP-expressing fibers in S1 and M1/M2 was 50% lower in TBI rats. This difference was more pronounced anteriorly than caudally, probably due to the fact that the thalamo-cortical relays from the ventral basal complex extend anteriorly [30,31]. The decreased expression of ChR2-eYFP-expressing thalamocortical projection terminals in the cortex of TBI rats may be related to neurodegeneration in the thalamus. The severity of thalamic neurodegeneration can vary from case to case, and thus affect the number of successfully transduced surviving neurons and consequently the density of the axonal terminals as discussed above. This hypothesis is supported by a previous report showing that degeneration of the thalamic relay neurons from the ventral basal complex (ventral posterior nucleus) of the thalamus resulted in pathology in the S1BF in the midline FPI model [22,23]. The ventral basal complex comprises the VPM and VPL thalamic nuclei, which project axonal relays to the S1BF [34]. These data suggest that the decrease in the cortical ChR2-eYFP-expressing fibers after TBI observed in our study is probably related to the severity of neurodegeneration in the thalamus.

To summarize, our data indicate that TBI induced by lateral FPI affects the thalamo-cortical axonal projections from the thalamic VPL/VPM nuclei, such that TBI animals exhibited a reduced density of thalamo-cortical axonal projection terminals in the cortex.

### 3.4. Thalamo-Cortical Network Demonstrates Signs of Hyperexcitability Following TBI

The optogenetics stimulation performed in this study was aimed at investigating whether the observed thalamo-cortical network structural reorganization (see above), particularly the VPL/VPM network that projects to lesioned S1, was functionally active. Furthermore, we aimed to determine whether the severely damaged thalamus contributes to the pathologic epileptogenic network in the perilesional cortex. A growing amount of functional or structural imaging data indicates extensive disruption of the thalamo-cortical network following TBI in both human TBI and animal models of TBI [9,10,11,12]. The evidence further suggests that the network around the perilesional area is epileptogenic [24,25]. In fact, we recently demonstrated that the onset zone of a pentylenetetrazol-induced seizure as indicated by functional magnetic resonance blood oxygenation level-dependent (fMRI-BOLD) responses was in the perilesional cortex of lateral FPI rats. These fMRI-BOLD responses, which spread bilaterally in the cortex, were also observed in the ipsilateral thalamus [24]. In another interesting study by Bragin and colleagues, pathologic high-frequency oscillations (pHFOs) were generated within the perilesional area (cortical areas within or adjacent to the lesion core) in lateral FPI rats. They further showed that pHFOs occurred in animals that later developed spontaneous seizures 1 to 3 months post-TBI [25]. These data suggest that ongoing plastic changes within the perilesional cortex underlie these TBI-related hyperactivity network responses. The present findings further corroborate this hypothesis by showing that optogenetic stimulation of a subset of neurons within the thalamic VPL/VPM thalamo-cortical network in TBI rats induced high-voltage, high-frequency activity within the beta frequency range that spread contralaterally compared with that in sham-injured control animals. In a similar experiment that we performed, stimulation of the VPL/VPM network in TBI rats resulted in an epileptic seizure (unpublished data). Baseline EEG showed no epileptiform activity during the 1-week follow-up prior to the optogenetics stimulation that may have contributed to this response in the TBI rats. The increased beta activity was reported in patients with post-traumatic stress disorder and was related to global cortical hyperexcitability, prolonged wakefulness, or attention disturbances [53]. The spread of the optogenetic stimulation response to the contralateral cortex corroborates the idea of global cortical hyperexcitability. However, further studies are needed to elucidate this hypothesis. It was previously demonstrated in a cortical photothrombotic stroke model that the surviving thalamo-cortical loop within the peri-stroke area can become hyperexcitable and generate seizures [54]. Considering the fact that the thalamus plays a critical role in relaying information to the cortex, the latter finding together with our finding suggests that the functionally altered activity of the surviving thalamo-cortical network contributes to creating an epileptogenic zone within the perilesional cortex. Moreover, thalamo-cortical network functional plasticity also has implications in other post-TBI comorbidities. In fact, Little and colleagues showed that damage to thalamic projection fibers as a result of diffused axonal injury accounts for variance in post-TBI executive function, attention, and memory [55]. The mechanism behind post-TBI thalamic network functional changes and these comorbidities requires further studies.

Taken together, our data indicate that the post-TBI thalamo-cortical network exhibits signs of hyperexcitability that may contribute to perilesional network plasticity.

### 3.5. Limitations and Future Direction

Although this study has provided some valuable insights into structural and functional alterations of thalamocortical axonal projections after severe TBI, there are some limitations to the study that should be acknowledged. First, it is important to note that though we observed marked changes in the beta frequency in TBI animals, we acknowledge that the low number of animals used in the functional studies does not give a true representation of the variability between the animals. Thus, caution should be employed when interpreting the data and more experiments are needed to further explore these findings. Second, though the structural difference between the TBI and sham or naïve rats was clear, we acknowledge that comparison of the structural data between ChR2-eYFP-transduced shams and TBI rats may have been more appropriate. Finally, for future studies, we propose that pre-injury transductions or the use of the Cre-recombination technique to mitigate the impact of the post-injury inflammatory response, thalamic atrophy, and neurodegeneration on transduction efficiency or targeting of the thalamic nucleus should be considered.

### 3.6. Conclusions

Our findings demonstrate that TBI caused by the lateral FPI model induces axonal plasticity in the VPL/VPM thalamo-cortical network and results in altered network activity that may be prone to hyperexcitation. These changes in network activity are associated with a reduced density of the thalamic axonal projection terminals in S1 because of the degeneration of thalamo-cortical relay neurons in the sensory thalamic nuclei, VPL/VPM. Moreover, the brain injury does not alter the laminar-specific targeting of the thalamic axonal projection terminals, but rather causes changes in the orientation dynamics of the fiber terminals, suggesting active post-injury re-organization of the axonal terminals in the cortex. These findings extend previous evidence suggesting structural and functional reorganization in the perilesional cortex following TBI and provide a structural basis for diffusion changes contributing to previously reported magnetic resonance imaging findings. Our data provide a starting point for studies exploring how changes in the connectivity and orientation dynamics of the axonal terminals in the perilesional cortex relate to the post-injury functional outcome.

## 4. Materials and Methods

### 4.1. Animals and Study Design

Adult male Sprague Dawley rats (350 to 450 g at the time of injury, from Harlan, Udine, Italy) were used in all experiments. Rats were housed in individual cages before and after TBI with free access to food and water. Animals were maintained on a 12-h light/12-h dark cycle with ambient temperature at 21 to 22 °C and 50 to 60% humidity. All animal procedures were approved by the Animal Ethics Committee of the provincial government of Southern Finland and carried out in accordance with the guidelines of the European Community Council Directives 2010/63/EU.

The rats were divided into two groups. The first group of rats was subjected to TBI (*n* = 11) using the lateral FPI model. The second group of rats underwent sham-injury (*n* = 2). The injury procedure is described below. One month after TBI, the ipsilateral thalamus was stereotaxically injected with a virus carrying genes for both enhanced yellow fluorescence protein (eYFP) and opsin channel rhodopsin-2 (ChR2/H134R; rAAV5/CaMKII-hChR2(H134R)-eYFP-WPRE-). After 3 months, an optical cannula was inserted into the ipsilateral thalamus and four cortical screw electrodes were implanted into the skull for video-electroencephalography (video-EEG) monitoring (see Supplementary Figure S2). Following a 1-week recovery period, the rats were optically stimulated with blue light. Thereafter, the animals were killed, and the brains were processed for histologic analysis. A separate group of naïve rats (*n* = 4) was used for the tracking of thalamocortical projections using phaseolus vulgaris-leucoagglutinin (PHA-L).

### 4.2. Induction of Traumatic Brain Injury

TBI was induced with lateral FPI as previously described [56,57]. Briefly, animals were anesthetized by intraperitoneal injection (6 mL/kg) of an anesthetic cocktail containing sodium pentobarbital (58 mg/kg), magnesium sulfate (127.2 mg/kg), propylene glycol (42.8%), and absolute ethanol (11.6%). The skull was exposed, and a 5 mm craniotomy was made between bregma and lambda on the left convexity, leaving the dura intact. The coordinate of the center of the craniotomy was AP −4.5 mm from bregma; ML 2.5 mm over the left cortex. TBI was induced with a fluid-percussion device (AmScien Instrument, Richmond, VA, USA). The force of the impact (fluid pressure) on the intact dura was 3.22 ± 0.01 atm. Durations of apnea and the impact-induced seizure-like behaviors were recorded. Control animals underwent the same anesthetic and surgical procedures, and were connected to the device, but no impact was induced.

### 4.3. Viral-Mediated Opsin Transduction and Optogenetic Stimulation in the Thalamus

#### 4.3.1. Viral Injection

At 1 month following TBI (*n* = 8) or sham-injury (*n* = 2) surgery, rats were injected with adeno-associated virus (AAV) carrying the plasmid construct rAAV5/CaMKII-hChR2(H134R)-eYFP-WPRE (University of North Carolina Gene Therapy Center, Chapel Hill, NC, USA). The AAV5 viral vector has a high transduction efficacy in neurons [58]. The CaMKII promoter limits the expression of ChR2-eYFP only to excitatory neurons. Moreover, the specificity of ChR2-eYFP on excitatory neurons has previously been reported by Zhang and colleagues [59]. In brief, animals were anesthetized (as described above) and a midline incision was made over the skull. A small craniotomy was performed on the left convexity at 3.12 mm posterior to bregma (bregma as reference) and 3.4 mm lateral to the midline according to the rat brain atlas [60]. The virus (0.5 μL) was injected into the ipsilateral thalamus 6.2 mm dorsoventrally, targeting the VPL and VPM thalamic nuclei. The virus was injected using a 5-μL Hamilton syringe and a 34-gauge needle at a flow rate of 0.1 μL/min. A micro pump (SYS-MICRO4, World Precision Instruments, Sarasota, FL, USA) was used to control the injection and flow rate. After injecting the virus, the needle was left in place for 5 min and then retracted slowly in two steps, with a 5-min interval between steps.

#### 4.3.2. Optical Cannula Placement and Implantation of Cortical Screw Electrodes

Three months after injection of the virus (see above), a fiber optic cannula (FT200EMT, Thorlabs; Newton, NJ, USA) made of an optical fiber (⌀ 200 µm) attached to a ceramic ferule (⌀ 2.5 mm) was inserted into the ipsilateral thalamus using the same coordinates as for the viral injection (anteroposterior: 3.12 mm, mediolateral: 3.4 mm, dorsoventral: 6.2 mm) with bregma as reference. The cannula was held in place with a small amount of dental acrylic to prevent movement during implantation of the electrodes. Four cortical screw electrodes were implanted into the skull. Two recording electrodes were implanted bilaterally into the skull over the somatosensory cortex and a ground and reference electrode were implanted into the skull over the occipital cortex. The head mount was held in place by dental acrylic. The animal was sutured and then allowed to recover in its home cage.

#### 4.3.3. In Vivo Optogenetic Stimulation and Video-EEG Monitoring

After a 1-week recovery period, the rats were connected to the video-EEG monitoring system [61]. Baseline EEG was collected for 1 week before the optical stimulations were started. To execute the optical stimulation, an optical ferrule patch cable (⌀ 200 µm) was connected to the fiber optic cannula using a cannula mating sleeve (ADAF1, Thorlabs). The other end of the optical patch cable was connected to a fiber-coupled LED source (470 nm) (M470F1) with an LED driver (DC2100, Thorlabs). The ChR2-eYFP-expressing neurons in the rats were stimulated with a 10-Hz, 30-ms pulse stimulation (30% duty cycle) for 5 s using a blue LED light (470 nm). Optical stimulations were performed twice each day (8-h interval) for 5 days. The power of the LED light at the cannula tip was 2.6 mW.

#### 4.3.4. Analysis of In Vivo EEG Recording

The 1-week baseline video-EEG recording was manually screened for epileptiform activity. Spectral analysis was performed on the 5-s EEG output from the optical stimulation on the ipsilateral electrode. Also, spectral analysis was performed on the baseline EEG recording of the ipsilateral electrode on a 5-s epoch. A period when the animal was awake was selected for the baseline analysis to match the same behavioral state as during the stimulations. The percentage of the voltage of each frequency band (alpha, beta, theta, and delta) during the stimulation period was normalized to the baseline then compared to each other. Comparison between the groups was not possible since only two ChR2-eYFP-sham rats were included.

### 4.4. Tissue Processing and Immunohistochemistry for the Validation of Ch2-eYFP Opsin Expression

#### 4.4.1. Tissue Processing

At the end of the experiment, animals were killed by transcardial perfusion. Briefly, animals were deeply anesthetized and then perfused with 0.9% NaCl solution (2 min, 3 mL/min), followed by cold (4 °C) 4% paraformaldehyde (30 min, 3 mL/min). The brains were removed from the skull, post-fixed in 4% paraformaldehyde for 2 h at 4 °C, and then cryoprotected in 20% glycerol prepared in 0.02 M potassium phosphate buffer solution (KPBS) for 24 to 48 h at 4 °C. Thereafter, the brains were frozen on dry ice and stored at –70 °C until further processing. The brains were cut into 30 µm thick coronal sections (1-in-10 series) using a sliding microtome. The first series of sections was stored in formalin and used for thionin staining to assess the tissue cytoarchitectonics. The remaining sections were stored in cryoprotectant tissue-collecting solution (30% ethylene glycol, 25% glycerol in 0.05 M sodium phosphate buffer) and stored at −20 °C.

#### 4.4.2. Immunostaining of ChR2-eYFP-Labeled Fibers

One series of sections from a 1-in-10 series was immunolabeled with antibodies against eYFP. First, the sections were washed in 0.02 M potassium phosphate buffer solution (KPBS) and then incubated in 1% hydrogen peroxide (H_2_O_2_) for 15 min at room temperature (RT). After washing in 0.02 M KPBS, the sections were incubated in a blocking solution containing 0.5% Triton-X 100 and 10% normal horse serum (NHS) in 0.02 M KPBS for 2 h at RT. Thereafter, the sections were incubated with a rabbit polyclonal antibody against green fluorescent protein (GFP) (1:16,000, Ab6556, Abcam) prepared in 2% NHS and 0.5% Triton-X 100 in KPBS, for two nights at 4 °C. The anti-GFP antibody was reported by the manufacturer to be reactive against all variants of *Aequorea Victoria* GFP. After washing in 2% NHS, the sections were incubated for 2 h at RT in a biotinylated horse antibody against Rabbit IgG (1:300, BA1100, Vector Laboratories, Burlingame, CA, USA). The sections were washed in 0.02 M KPBS and then incubated for 1 h at RT with avidin-biotin complex (ABC) solution (1:100, Vector Laboratories). After washing in 0.02 M KPBS, they were recycled into the secondary antibody (45 min, RT) and then into the ABC solution (30 min, RT). Next, the sections were washed in 0.02 M KPBS, and then incubated with 0.1% 3′,3′-diaminobenzidine (Pierce Chemicals, Rockford, IL, USA) solution containing 0.04% H_2_O_2_ and 0.02 M KPBS for 3 min at RT. Following extensive washing in 0.02 M KPBS (two times, 10 min each) and 0.1 M phosphate buffer (once, 10 min), the sections were mounted on gelatin-coated slides and dried overnight. The reaction product was intensified with osmium (OsO4; #19170; Electron Microscopy Sciences, Hatfield, PA, USA) and thiocarbohydrazide (#21900; Electron Microscopy Sciences).

### 4.5. Identification and Analysis of ChR2-eYFP Axonal Projection Terminals

#### 4.5.1. Outlining the Cortical Layers and Identification of Axonal Projection Cortical Targets

High-resolution panoramic brightfield images of the ipsilateral cortex of thionin-stained sections, and darkfield images of the ipsilateral cortex of the adjacent eYFP-immunolabeled sections were captured with the Zeiss imager2 microscope (Carl Zeiss AG, Germany) using the X5 objective. The images of the thionin-stained sections were uploaded into Photoshop and the whole section and cortical layers were outlined on a transparent Photoshop layer that was placed on top of the image. To identify the different cortical layers on the eYFP-immunolabeled sections, the outlines from the thionin-stained sections were superimposed onto the darkfield images of the adjacent eYFP-immunolabeled sections (See Figure 4). From the outlined cortical layers on the images of the eYFP-immunolabeled sections, we then analyzed the location and density of the axon terminals as well as their laminar distribution and orientation within the perilesional somatosensory and motor cortices (see below).

#### 4.5.2. Analysis of the Density of ChR2-eYFP-Expressing Fibers

High-resolution darkfield images of the ipsilateral hemisphere of sections that were eYFP-labeled (rostrocaudally about eight to nine sections per case) were captured with the Zeiss imager2 (see above). Using the adjacent Nissl-stained sections, the cortical layers and cortical areas (according to the rat atlas by Paxinos and Watson, 2007) were outlined as described above. The outlined darkfield images were imported into ImageJ software as tiff files. The software was scaled and the regions of interest (ROI) were further outlined from the thionin-stained sections. Due to injury-related damage that ruined the cytoarchitecture, we also referred to the rat brain atlas [60] to outline the ROI. The ROI included the ipsilateral primary and secondary motor cortex (M1 and M2, respectively), and S1. The image was then converted into an 8-bit grayscale image and threshold to select all the stained fibers. From the analysis menu, the area of the eYFP-labeled ChR2-expressing fibers was estimated. Additionally, the density of the eYFP-labeled fibers was estimated as a fraction of the total area of the selected ROI. The density was estimated separately in S1 and M1/M2. The resulting data output was transferred to Excel for further analysis.

### 4.6. Confirmation of the ChR2-eYFP-Traced Tracts and Cortical Targets Using Phaseolus Vulgaris-Leucoagglutinin (PHA-L)

#### 4.6.1. Injection of PHA-L

To confirm and compare the cortical targets of the ChR2-eYFP thalamic axonal projection terminals, the anterograde tracer PHA-L was injected into the thalamus of the left hemisphere of naïve rats using the same coordinates as described for ChR2-eYFP transduction (see above), targeting the VPL/VPM thalamic nuclei. Briefly, animals were anesthetized as described above and a midline incision was made. A small craniotomy was made on the left convex using the same coordinates for the viral injection described above (anteroposterior: 3.12 mm, mediolateral: 3.4 mm, and dorsoventral: 6.2 mm). PHA-L was injected into the thalamus using the protocol described by Kemppainen and colleagues [62]. At 10 days after the injection, PHA-L-injected rats were killed by transcardial perfusion with 4% paraformaldehyde as described above (see tissue processing).

#### 4.6.2. PHA-L Immunohistochemistry

One series of sections from a 1-in-5 series (30-µm thick sections) was immunolabeled to identify PHA-L immunoreactive (ir) fibers. The immunolabeling protocol was the same as for the ChR2-eYFP labeling, except that the primary antibody was rabbit anti-PHA-L (1:8000 and the secondary antibody was biotinylated goat anti-rabbit IgG (1:200. #BA-1000, Vector Laboratories) [62]. The reaction product was intensified with osmium (OsO4; #19170; Electron Microscopy Sciences, Hatfield, PA, USA) and thiocarbohydrazide (#21900; Electron Microscopy Sciences).

### 4.7. Nematic Tensor-Based Concept (FibrilTool) Analysis of Fiber Orientation and Anisotropy

The “FibrilTool” in ImageJ^®^ software was used to analyze the orientation and anisotropy of the thalamo-cortical projection terminals in layer V of S1 [63]. Darkfield images of the eYFP (ChR2-eYFP) and PHAL-labeled sections with the cortical layer V outlined (as described above) were uploaded into a scaled ImageJ^®^ software as tiff files. The ROI in S1 was selected using the polygon tool (see Figure 6). The average orientation and anisotropy within the selected ROI were estimated by clicking on the fibril tool. Approximately six to eight sections rostrocaudally (bregma level +0.4 to −2.4) were analyzed. The orientation is estimated based on the x-axis of the image. All the cortical images were aligned in the same way for the analysis (see Figure 6). The resulting output was then transferred to Excel for further analysis.

### 4.8. Statistical Analysis

The IBM SPSS statistic25 package and R-studio (v1.1.463) were used for statistical analysis [64]. A nonparametric Mann-Whitney U test was used to determine the difference in anisotropy between TBI and controls in all the sections analyzed. A mixed-effect model analysis was used to assess rostrocaudal differences in anisotropy between TBI and controls. The R package circular (v0.4-93) was used to compute the directional statistics [65]. Watson’s Two-Sample Test of Homogeneity was used to determine differences in the orientation of the projection terminal in cortical layer V between TBI and controls. One-way ANOVA followed by multiple comparison was used to determine differences in the percentage of voltage change between the frequency bands in TBI rats following optical stimulation. Comparison of the percentage of voltage change between the groups was not possible since only two ChR2-eYFP-sham rats were included. Data are presented as mean ± standard error of the mean (SEM). A *p*-value < 0.05 was considered statistically significant.

## Data Availability

The data presented in this study are available on request from the corresponding author. The data are not publicly available due to privacy.

## Supplementary Materials

The following are available online at https://www.mdpi.com/article/10.3390/ijms22126329/s1.
