# Supplementary material for: Reorganization of Thalamic Inputs to Lesioned Cortex Following Experimental Traumatic Brain Injury
Source: Int J Mol Sci. 2021 Jun 13;22(12):6329. doi: 10.3390/ijms22126329 (PMC8231773; doi:10.3390/ijms22126329)
Supplement: Supplementary file 1 [file ijms-22-06329-s001.zip › ijms-1220517-supplementary.pdf]

Supplementary Materials

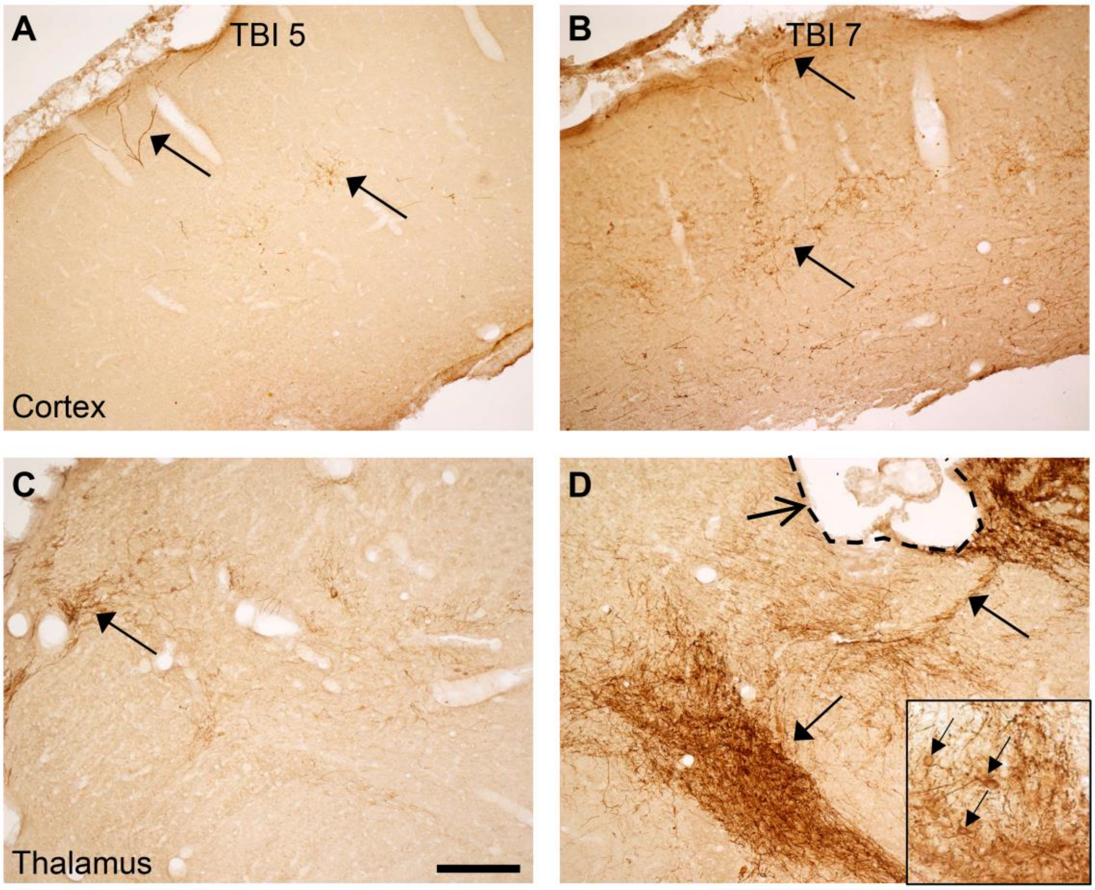

Supplementary figure S1. Photomicrograph demonstrating the expression of ChR2-eYFP in the cortex and thalamus of two traumatic brain injured (TBI) rats that showed no EEG response during optogenetic stimulation. (**A, C**) the cortex and thalamus (filled arrows) respectively of case number 5 (TBI5). This case (out of 4 TBI non-responding) had the lowest expression of ChR2-eYFP. (**B, D**) TBI case number 7 (TBI7) had the most expression (of the 4 TBI non-responding cases) of ChR2-eYFP in the cortex and the thalamus (filled arrows). Note the tip of the optical cannula in the thalamus (dotted black line, open arrowhead). Scale bar equals 200µm.

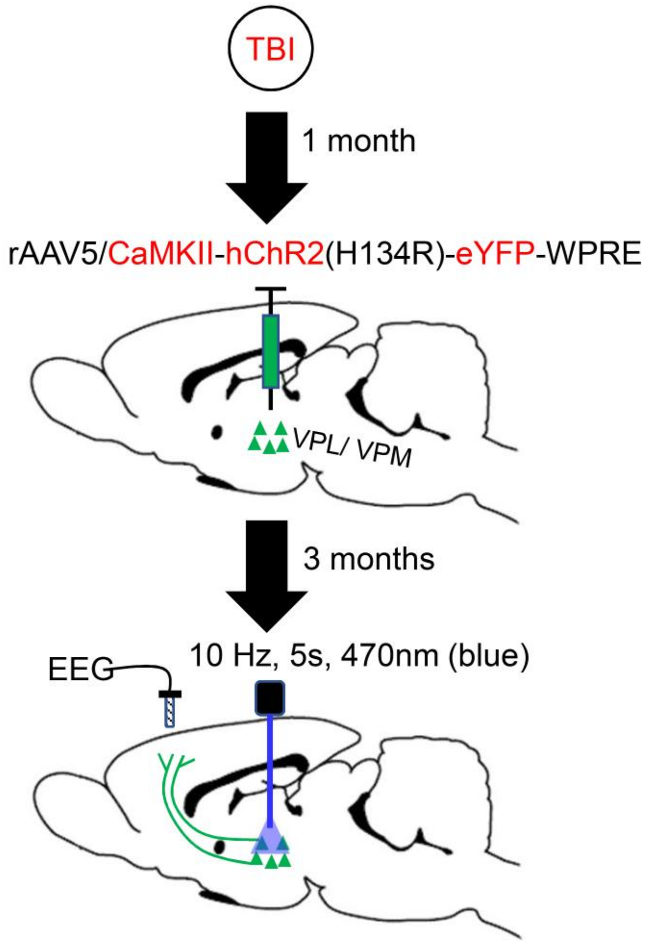

Supplementary figure S2. Study design. Rats were injured using the lateral fluid percussion brain injury model. Sham-operated controls went through same surgical procedure but did not receive the injury. At 1-month post-injury, rats were injected into the thalamus targeting the thalamic VPL/VPM nuclei with an AAV carrying the transgene for the ChR2 opsin with eYFP as a reporter. Three months after the transfection, the rats were implanted with an optical cannula and four cortical screw electrodes. Following 1 week of baseline video-EEG recording, the rats were optogenetically stimulated twice daily (8-h intervals) with a blue light (470nm) for 5 days and the output recorded. The rats were killed the day after the last stimulation by transcardial perfusion with 4% paraformaldehyde and the brains processed for histologic and immunohistochemical analysis.
